# Age-specific MRI patterns in pediatric epilepsy: insights from a sudanese cohort and implications for low-resources settings

**DOI:** 10.1186/s12880-026-02549-z

**Published:** 2026-07-02

**Authors:** Yousif Mohamed, Abubaker Babiker, Hind Elamin, Esra Abdalla

**Affiliations:** 1 Facutly of Medicine, University of Khartoum, Khartoum, Sudan; 2 Faculty of Medicine, Gadarif University, Gadarif, Sudan; 3 College of Nursing and Health Science, Jazan University, Jazan, Saudi Arabia

**Keywords:** Pediatric epilepsy, MRI findings, Mesial temporal sclerosis, Sudan

## Abstract

**Objective:**

Epilepsy is a global health problem affecting the quality of life of many children. Neuroimaging, particularly Magnetic Resonance Imaging (MRI), plays a crucial role in precisely identifying epileptogenic foci that are potentially amenable to surgical resection for a possible cure. This study aims to evaluate the diagnostic yield of MRI in a Sudanese pediatric epilepsy cohort and to identify age-specific neuroimaging patterns to optimize resource allocation in low-income settings.

**Methods:**

A descriptive cross-sectional study enrolled 100 pediatric patients (≤ 17 years) diagnosed with epilepsy from June 2023 to June 2024. Data on age, gender, and MRI findings were collected and analyzed using SPSS v26.0. Chi-square tests assessed univariate associations, and multivariate logistic regression evaluated the combined impact of age and gender on abnormal MRI findings, with significance set at *P* < 0.05.

**Results:**

Of 100 patients (55% male, 45% female), 87% were aged 1–12 years. MRI abnormalities were detected in 31%, with mesial temporal sclerosis (35.4%) most frequent, followed by brain atrophy and periventricular leukomalacia (16% each). Age was significantly associated with abnormalities (*P* = 0.002), with higher odds in < 1-year-olds (OR = 9.35, 95% CI: 1.67–52.41, *P* = 0.011) and 13–17-year-olds (OR = 14.20, 95% CI: 1.47–137.32, *P* = 0.021) compared to 1–6-year-olds. Specific findings varied by age: periventricular leukomalacia in < 1-year-olds, atrophy in 1–6 years, mesial temporal sclerosis in 7–12 years, and tumors in 13–17 years (*P* < 0.002). Gender showed no significant effect (OR = 0.74, 95% CI: 0.32–1.71, *P* = 0.480).

**Conclusion:**

Approximately one-third of children with epilepsy showed MRI abnormalities, most commonly mesial temporal sclerosis. Abnormal MRI results were notably linked to patient age, particularly those under one year old and between 13 and 17 years old. Age-specific MRI protocols could enhance diagnostic efficiency in low-resources settings. These findings support for prioritized neuroimaging in infants and adolescents, informing public health strategies to address Sudan’s epilepsy burden.

**Clinical trial number:**

Not applicable.

## Background

Epilepsy is a common neurological condition affecting over 50 million people worldwide, with 85% of those cases occurring in developing countries [1]. It is characterized by recurrent seizures caused by abnormal electrical activity in the brain, affecting individuals across all demographics. The prevalence of active epilepsy is estimated at 4–10 per 1,000 individuals in the general population and 6–10 per 1,000 in developing countries [1]. Half of all epilepsy cases begin in childhood or adolescence, and approximately 70–80% of patients can lead normal lives with proper treatment [1].

Seizures can be categorized as focal, affecting one hemisphere of the brain, or generalized, involving both hemispheres, and may result in a loss of consciousness or altered awareness. The causes of epilepsy can be classified as symptomatic (secondary to a brain injury) or idiopathic [2]. According to the 2014 International League Against Epilepsy (ILAE) definition, epilepsy is diagnosed when an individual experiences two unprovoked seizures more than 24 h apart, with at least one seizure having a high probability (≥ 60%) of recurrence within a decade [3]. A diagnosis is considered resolved if the individual has been seizure-free for 10 years while off medication for 5 years or beyond the age-related risk for seizures [4].

Globally, the incidence of epilepsy varies: there are 33.3–82 new cases per 100,000 people each year in developed countries and up to 187 new cases per 100,000 in developing countries [5, 6]. The highest incidence occurs in the first year of life (102 per 100,000) and among children aged 1–12 years. After that, the incidence decreases to 21–24 cases per 100,000 among those aged 11–17, and remains relatively stable from age 25, with a slight male predominance [5]. Mortality rates are 2–4 times higher than average, and 5–10 times higher in children, with global rates of 2.7–6.9 deaths per 1,000 children per year; sudden unexpected death in epilepsy (SUDEP) accounts for 1.1–2 deaths per 10,000 children each year [7, 8]. Magnetic resonance imaging (MRI), along with electroencephalogram (EEG) tests, are crucial for visualizing brain anatomy and identifying structural lesions that are necessary for accurate diagnosis, treatment planning, and prognosis in pediatric epilepsy [9].

Structural lesions associated with epilepsy can be acquired (for example, hippocampal sclerosis or brain injuries due to infection or trauma) [10, 11] or congenital (for example, malformations of cortical development, such as lissencephaly or polymicrogyria) [12, 13]. Cerebral tumors, including dysembryoplastic neuroepithelial tumors (DNET), are responsible for 1–3% of new-onset seizures in children and account for 10% of tumors presenting with epilepsy [14, 15]. Other tumors, such as gangliogliomas, may be associated with cortical dysplasia [16]. Vascular complications, occurring in 20–45% of arteriovenous malformations (AVMs) and cavernous angiomas, can also lead to seizures, which may be detected on MRI with susceptibility-weighted imaging (SWI) [17, 18]. In Sub-Saharan Africa, including Sudan, limited access to neuroimaging hampers the diagnosis and treatment of epilepsy [15]. The prevalence of pediatric epilepsy in Sudan is higher than in Europe, with 53% of affected individuals surviving beyond five years—62% with generalized epilepsy, 31% with focal epilepsy, and 7% with syndromic epilepsy—indicating a significant public health crisis stemming from healthcare disparities [19].

While MRI is standard globally, to our knowledge, this is the first study to document age-specific MRI findings in a Sudanese pediatric epilepsy cohort. These data provide actionable insights for similar low-resource settings, where MRI access is limited to 1.3 scanners per million population. Our findings can guide targeted neuroimaging to optimize resource allocation.

## Methodology

### Study design

This study employed a descriptive cross-sectional hospital-based design to evaluate MRI findings in pediatric patients diagnosed with epilepsy.

### Study area

The study was conducted at Al-Daman Hospital in Marawi, located in the Northern state of Sudan. Established in 2016, the hospital has grown to become a key healthcare provider, offering various medical services. It is a tertiary referral centre serving the Northern state of Sudan and surrounding regions. The facility has a capacity of approximately 250 beds, distributed across different specialties, including pediatrics, surgery, medicine, obstetrics, and gynecology. The radiology department is equipped with a variety of imaging modalities, such as ultrasound, fluoroscopy, computed tomography (CT), and MRI. It is staffed by four radiologists and three well-trained radiologic technologists.

### Study duration

The study was conducted over a one-year period, from June 2023 to June 2024.

### Study population

The study population comprised pediatric patients clinically diagnosed with epilepsy who underwent MRI examinations during the study period. MRI was not performed on all children with epilepsy in the hospital due to limited scanner availability and cost. Patients included in this study were those referred for MRI by treating neurologists based on clinical judgement (typically including drug‑resistant epilepsy, focal seizures, or abnormal neurological examination). However, the specific indications for MRI referral were not systematically obtained. CT imaging was available at the hospital but was not routinely performed prior to MRI in this cohort.

### Inclusion criteria

- Pediatric patients aged 17 years or less.
- Cases with complete and accurate medical records.

### Exclusion criteria

- Patients with MRI images of inadequate quality due to motion artifacts.
- Patients with a history of head trauma or previous neurosurgical intervention.
- Patients with severe hydrocephalus (or other conditions causing extensive parenchymal distortion, such as large cephalic cysts), because the associated periventricular edema, ventricular enlargement, and parenchymal thinning can obscure or mimic true epileptogenic lesions. In our resource‑limited setting without advanced processing, such cases were excluded to avoid diagnostic confounding.

### Sample size and sampling technique

A total coverage sampling approach was employed, enrolling all accessible pediatric patients with epilepsy diagnosed at Al-Daman Hospital from June 2023 to June 2024 who met the inclusion criteria. Of 112 consecutive pediatric epilepsy patients who underwent MRI during the study period, 12 were excluded: 7 due to motion artefacts compromising image quality, 3 with a history of prior neurosurgical intervention, and 2 with severe hydrocephalus. The final analysed sample was 100 patients. The sample size was also justified on the basis of power calculations to detect MRI abnormalities on the assumption of a 30% prevalence, as per local studies like Apolot et al. [15], at a confidence level of 95% and a margin of error of 10%, which required at least 81 participants. Considerations in a single-center, resource-limited setup with an epilepsy patient load of approximately 120–150 per annum (hospital records), 100 was both statistically adequate and pragmatically feasible, covering most of the eligible cases within the study period.

### Data collection tools and methods

Data collection was conducted by the principal investigator using a structured data collection sheet. The collected data included:

- Age.
- Gender.
- MRI findings.
- Clinical data on age at seizure onset, history of febrile seizures, status epilepticus, CNS infections or perinatal insults were not systematically collected.

### Local MRI protocol

The MRI scans were performed on a 1.5T scanner (Siemens, Germany) using a standardized epilepsy protocol, including:

- Standard thin-slice (3 mm) axial and sagittal T1-weighted sequences.
- Axial and coronal (3 mm) T2‑weighted sequences, with coronal slices perpendicular to the hippocampal axis.
- Axial and coronal Fluid-Attenuated Inversion Recovery (FLAIR) sequences parallel to the temporal lobes.
- Axial diffusion-weighted imaging (DWI), apparent diffusion coefficient (ADC) map, and gradient-weighted sequences.
- All scans were independently reviewed by two consultant radiologists blinded to clinical data; disagreements were resolved by consensus. No genetic testing was performed.

### Study variables

#### Independent variables

- Age.
- Gender.

#### Dependent variables

- MRI findings.

### Data analysis

Data were analyzed using Statistical Package for Social Sciences (SPSS) version 26.0. Descriptive statistics were used to describe MRI findings, gender, and age. Chi-square test was used to analyze univariate associations of MRI findings with independent variables (gender, age) at p-value < 0.05. For the assessment of combined effects of gender and age on the likelihood of abnormal MRI findings, a multivariate logistic regression analysis was used. The outcome variable was dichotomous (MRI-positive vs. MRI-negative), and gender (male vs. female) and age (categorized as < 1, 1–6, 7–12, 13–17 years) were predictors. Odds ratios (OR) with 95% confidence intervals (CI) were estimated, and model fit was assessed using the Hosmer-Lemeshow test. Given the limited sample size and number of abnormalities (31 MRI‑positive cases), all results – particularly those with p‑values between 0.01 and 0.05 – should be interpreted with caution.

## Results

A total of 100 pediatric epilepsy patients were enrolled: 55% male, 45% female, with 87% aged 1–12 years (44% aged 1–6, 43% aged 7–12). MRI abnormalities were detected in 31% of cases, with mesial temporal sclerosis (MTS) as the most common (11 cases, 35.4%), followed by brain atrophy and periventricular leukomalacia (5 cases each, 16%), neuronal migration disorders (3 cases, 9.6%), tumors and leukodystrophy (2 cases each, 6.4%), vascular malformations and cystic encephalomalacia (1 case each, 3.2%). Among MTS cases, 54.5% were right-sided, 45.5% left-sided; brain atrophy was predominantly hemi-atrophy (80%); and neuronal migration disorders showed focal cortical dysplasia (66%) over lissencephaly (33%) (Table 1). Among the 4 patients with hemi‑atrophy, none showed the characteristic imaging features of Rasmussen’s encephalitis. However, early or atypical cases cannot be excluded without serial imaging or EEG correlation. Furthermore, due to small numbers (e.g., tumors *n* = 2, vascular malformations *n* = 1), no statistical comparisons were performed for these rare findings, and they are reported descriptively.

**Table 1:** Distribution and characteristics of some abnormal MRI findings among epileptic pediatric patients

MRI abnormalities (*N*)		Frequency	Percentage of *N*
Mesial temporal sclerosis (11)	Right sided	6	54.6%
	Left sided	5	45.4%
Brain atrophy (5)	Hemi-atrophy	4	80%
	Global	1	20%
Neuronal migration disorders (3)	Focal Cortical Dysplasia	2	66.6%
	Lissencephalic pattern	1	33.4%

Univariate analysis via Chi-square revealed a significant association between MRI abnormalities and age (*P* = 0.002), with higher rates in < 1-year-olds (75%) and 13–17-year-olds (80%) compared to 1–6 (22.7%) and 7–12 years (25.6%) (Table 2). Specific abnormalities varied by age: periventricular leukomalacia dominated in < 1-year-olds, brain atrophy in 1–6 years, MTS in 7–12 years, and tumors in 13–17 years (*P* < 0.002) (Table 3). Gender showed no significant association with MRI findings or abnormality distribution (*P* = 0.414) (Table 4).

**Table 2:** The association between MRI findings and age of patients

Age in years (N)	Percentage	MRI-negative (% of N)	MRI-positive	P- value
< 1 (8)	8%	2 (25%)	6 (75%)	**0.002**
1–6 (44)	44%	34 (77.3%)	10 (22.7)	
7–12 (43)	43%	32 (74.4%)	11 (25.6%)	
13–17 (5)	5%	1 (20%)	4 (80%)	

**Table 3:** Association between MRI abnormalities and age of patients

MRI abnormalities (N)	< 1 year ( % of N)	1–6	7–12	13–17	P
Mesial temporal sclerosis (11)	0 (0.0%)	1 (9.1%)	10 (90.9%)	0 (0.0%)	**< 0.002**
Neuronal migration disorders (3)	0 (0.0%)	3 (100%)	0 (0.0%)	0 (0.0%)	
Tumors (2)	0 (0.0%)	0 (0.0%)	0 (0.0%)	2 (100%)	
Vascular malformations (1)	0 (0.0%)	0 (0.0%)	0 (0.0%)	1 (100%)	
Cystic encephalomalacia (1)	0 (0.0%)	0 (0.0%)	0 (0.0%)	1 (100%)	
Periventricular leukomalacia (5)	5 (100%)	0 (0.0%)	0 (0.0%)	0 (0.0%)	
Brain atrophy (5)	0 (0.0%)	5 (100%)	0 (0.0%)	0 (0.0%)	

**Table 4:** the association between MRI abnormalities and gender of patients

MRI abnormalities (N)	Male (% of N)	Female (% of N)	P value
Mesial temporal sclerosis (11)	5 (45.4%)	6 (54.6%)	**0.414**
Neuronal migration disorders (3)	2 (66%)	1 (33%)	
Tumors (2)	2 (100%)	0 (0.00%)	
Vascular malformations	0 (0.00%)	1 (100%)	
Cystic encephalomalacia	1 (100%)	0 (0.00%)	
Periventricular leukomalacia	4 (80%)	1 (20%)	
Brain atrophy	4 (80%)	1 (20%)	

Multivariate logistic regression further clarified these relationships (Table 5). Adjusting for gender, age remained a significant predictor of MRI abnormalities. Compared to the 1–6-year reference group (lowest abnormality rate), the odds of an abnormal MRI were significantly higher for < 1-year-olds (OR = 9.35, 95% CI: 1.67–52.41, *P* = 0.011) and 13–17-year-olds (OR = 14.20, 95% CI: 1.47–137.32, *P* = 0.021). The 7–12-year group showed no significant difference (OR = 1.18, 95% CI: 0.47–2.97, *P* = 0.727). Gender was not a significant predictor (OR = 0.74, 95% CI: 0.32–1.71, *P* = 0.480). The Hosmer-Lemeshow test indicated good model fit (*P* = 0.682).

**Table 5:** Multivariate logistic regression analysis of factors associated with abnormal MRI findings

Variable		Odds ratio	95% CI	*P*- value
Age (ref : 1–6 years )	< 1	9.34	1.67–52.41	0.011
	7–12	1.18	0.47–2.97	0.727
	13–17	14.2	1.47–137.32	0.021
Gender (ref : Male) Female		0.74	0.32–1.71	0.480

Hosmer-Lemeshow: *P* = 0.710 (good fit)

## Discussion

Epilepsy is a complex neurological disorder, and its management depends on accurate diagnostic imaging. Our investigation aimed to analyze the MRI findings in children with epilepsy, identify the etiological factors, and explore the relationship between imaging results and clinical characteristics. These findings shed light on the significant role of MRI in pediatric epilepsy across age groups.

Our study demonstrated that age plays a significant role in pediatric epilepsy. The majority of patients (87%) were within the 1–12 year age range, with 44% in the 1–6 year group and 43% in the 7–12 year group, and a mean age of presentation at approximately 8.79 years. This aligns with findings from other studies, such as those by Pal et al. [20] and Mundhe et al. [21], which reported a higher prevalence of epilepsy in the 6–12 year age group, and Alkhedir et al. [6] in Sudan, which observed a mean age of 8.4 ± 4.7 years in 350 pediatric patients.

Furthermore, age was significantly associated with the presence and type of MRI abnormalities. Children under one year and those aged 13–17 years showed a higher likelihood of abnormal MRI findings (*P* = 0.002). Specific abnormalities were observed across age groups: periventricular leukomalacia in children under one year, brain atrophy in those aged 1–6 years, MTS in the 7–12 year group, and tumors in adolescents aged 13–17 years (*P* < 0.002). These findings are consistent with those of Amirsalari et al. [22] in Iran, who also demonstrated a significant association between Abnormal MRI findings and patient age (*P* < 0.05). These findings emphasize the importance of considering age-specific risk factors and potential etiologies in pediatric epilepsy, such as perinatal insults in younger children and genetic factors in late-onset cases.

This study observed a male predominance among patients, with 55% being male and 45% female (male: female ratio of 1.2:1), consistent with findings from previous studies in Sudan [19, 23], Egypt [1], and Saudi Arabia [9], which reported similar male-to-female ratios. While this male predominance is consistently observed, the underlying reasons remain largely unclear. Some research suggests that hormonal factors and a higher risk of injury-related seizures in males may contribute to this gender disparity [24, 25]. Furthermore, the male predominance in some abnormalities may be the result of limited sample sizes rather than being a reflection of biological tendencies.

Age significantly influenced MRI findings, with multivariate analysis confirming higher odds of abnormalities in < 1 year (OR = 9.35) and 13–17 years (OR = 14.20) compared to 1–6 years age group. This refines the univariate findings (*P* = 0.002), suggesting age-specific etiologies: perinatal insults in infants (e.g., periventricular leukomalacia) and tumors in adolescents. These align with Amirsalari et al. [22] and highlight age as a critical diagnostic factor. Gender’s lack of effect (OR = 0.74, *P* = 0.480) contrasts with Yapici O et al. [26] but supports broader consistency in MRI findings across genders.

Abnormal MRI findings were observed in 31% of cases, consistent with the findings of several previous studies. Amirsalari et al. [22], Mathur et al. [27], Pal et al. [20], Yapici et al. [26], Sahdev et al. [28], Kalnin et al. [29], and Durá-Travé et al. [30] reported MRI positivity rates ranging from 28.5% to 39%, aligning with our study findings. Furthermore, significantly higher rates of MRI abnormalities were reported in the studies by Apolot et al. [15] and Ojaswi et al. [31], with lesion detection rates of 74.2% and 82%, respectively.

Our study identified MTS as the most frequent MRI-detected etiology (35.4%), followed by brain atrophy and periventricular leukomalacia (16% in both). Other findings included vascular malformations and cystic encephalomalacia (3.2% in each). These findings exhibit some variability compared to other studies.

For instance, Apolot D et al. [15] observed hippocampal sclerosis as the most common abnormality, while Youssf et al. [1] reported brain atrophy, brain tumors, and cortical dysplasia as the most frequent findings. Raafat et al. [2] identified white matter disease as the leading cause, followed by MTS and hydrocephalus. Alyoubi et al. [9] found developmental anomalies to be the most common abnormality, followed by hypoxic-ischemic injury, vascular abnormalities, metabolic disorders, and infections. Amirsalari et al. [22] observed brain atrophy, increased white matter signal intensity, benign cysts, brain tumors, and vascular abnormalities. Mathur et al. [27] found inflammatory granulomas to be most common, while Nilkanth L et al. [20] reported cortical gliosis as the most frequent abnormality among positive cases. Finally, Yapici O et al. [26] identified previous parenchymal damage as the most common etiologic finding. These variations in MRI findings across studies are likely influenced by several factors, including geographical location, ethnicity, genetic and biological factors, study population characteristics, sample size, and sampling methods.

This high proportion of MTS is consistent with findings from other low and middle-income countries. In a community‑based study in rural China, Davagnanam et al. [32] reported that 38% of people with chronic epilepsy had potentially epileptogenic lesions, with MTS among the commonest findings. In Bhutan, Bruno et al. [33] found MTS in 53% of participants, exceeding neurocysticercosis and congenital malformations. In Kenya, Samia et al. [34] reported hippocampal sclerosis in 2.8% of children, though encephalomalacia and cerebral atrophy were more common in that cohort. More recently, Kariuki et al. [35] identified brain abnormalities in over 60% of people with active convulsive epilepsy in Kenya and South Africa, with MTS among the predominant findings.

Furthermore, this predominance is notably higher than reported in many European and North American paediatric epilepsy series, where focal cortical dysplasia often ranks first. These comparisons suggest that the high MTS burden observed in Sudan reflects a broader pattern across resource‑limited settings, likely driven by preventable risk factors such as prolonged febrile seizures, CNS infections, and delayed access to care. Consequently, our findings should not be generalized uncritically to other paediatric epilepsy populations; they highlight the need for targeted diagnostic and preventive strategies.

In addition to age, other clinical factors were reported to influence the diagnostic yield of MRI in pediatric epilepsy. Recently, Winston et al. [36] recommended MRI for all infants with new‑onset epilepsy, particularly those with focal features or abnormal neurological examination. Hourani et al. [37] found that the presence of developmental delay and specific seizure types (focal seizures, epileptic spasms) were independently associated with a higher likelihood of detecting an epileptogenic lesion on MRI. Similarly, Coryell et al. [38] reported that imaging abnormalities were most common in children with focal seizure semiology (40%), spasms (47%), or unclear semiology (42%). In our cohort, these clinical data were not systematically collected. Future prospective studies in Sudan should integrate detailed seizure phenotyping, developmental assessment, and EEG correlation to refine MRI algorithms.

This study has several important limitations. First, it is a single‑centre, hospital‑based cohort, introducing selection bias and limits generalizability. Second, the sample size (*n* = 100, with only 31 MRI‑positive cases) limits statistical power, resulting in wide confidence intervals; thus, our results should be interpreted cautiously. Third, we did not collect data on key clinical variables known to influence MRI findings, including: age at seizure onset, seizure type (focal vs. generalised), presence of developmental delay, neurological examination findings, perinatal history (e.g., prematurity, birth asphyxia), history of febrile status epilepticus or CNS infections, use of anti‑seizure medications, or whether epilepsy was new‑onset or drug‑resistant. These data were not systematically available in our retrospective chart review. Fourth, no genetic or metabolic testing was performed. Fifth, the study lacked a formal IRB number (though ethical approval was obtained per local standards). Lastly, we did not have access to EEG data for correlation with MRI findings.

In Sudan and other similar contexts where MRI scanners are scarce (< 2 per million population) and many families cannot afford imaging, our findings support a targeted approach. Children under 1 year and adolescents aged 13–17 years had the highest odds of abnormal MRI (OR 9.35 and 14.20, respectively), regardless of gender. Therefore, where universal MRI is impossible, priority should be given to these age groups. MTS is a known surgical target via temporal lobectomy. However, epileptic surgery is not currently performed in Sudan due to the lack of specialized neurosurgical and intraoperative EEG facilities. Our findings can inform health system planning by quantifying the potential surgical burden and supporting the development of referral pathways to regional or international epilepsy surgery centres.

Future prospective studies in Sudan should integrate standardized clinical, EEG, and MRI data collection with defined protocols for metabolic and genetic testing where feasible. Such an approach would enable subgrouping – for example, distinguishing MRI patterns in focal vs. generalised epilepsy, or identifying imaging biomarkers for treatable inflammatory/metabolic conditions.

## Conclusion

This study emphasizes MRI’s crucial role in pediatric epilepsy, revealing abnormal findings in about one-third of cases, mainly mesial temporal sclerosis. Age significantly influenced MRI abnormalities, while gender did not. Early MRI use is recommended for diagnosis, with protocol optimization to improve accuracy. Future research combining MRI with detailed clinical phenotyping, EEG, and metabolic/genetic testing is needed to enhance etiological subgrouping and improve diagnostic accuracy in low‑resource settings.

## Data Availability

All data supporting the findings of this study are available within the paper and its Supplementary Information.

## Abbreviations

ADC: Apparent diffusion coefficient
AVM: Arteriovenous Malformations
CT: Computed Tomography
CI: Confidence interval
DNET: Dysembryoblastic Neuroepithelial Tumors
DWI: Diffusion-weighted imaging
EEG: Electroencephalogram
FLAIR: Fluid Attenuation Inversion Recovery
ILAE: International League Against Epilepsy
MRI: Magnetic Resonance Imaging
SPSS: Statistical Package For Social Studies Program
SUDEP: Sudden Unexpected Death In Epilepsy
SMSB: Sudan Medical Specialization Board
SWI: Susceptibility-weighted Imaging
